# Correction to ‘Intermediacy of publications’

**DOI:** 10.1098/rsos.201326

**Published:** 2020-09-02

**Authors:** Lovro Šubelj, Ludo Waltman, Vincent Traag, Nees Jan van Eck

*R. Soc. Open Sci.* **7**, 190207. (Published 15 January 2020) (doi:10.1098/rsos.190207)

The below sentence has been added to the Introduction.

Preliminary work on the intermediacy was presented in [23].

**Changes to the reference list**

The following reference should, therefore, be added to the reference list.

23. Šubelj L, Waltman L, Traag V, van Eck NJ. 2019 Intermediacy of publications. In *Proc. 17th Int. Conf. on Scientometrics & Informetrics ISSI ’19*, Rome, Italy, 2 September, pp. 1288–1300. Leuven, Belgium: ISSI. See http://issi-society.org/publications/issi-conference-proceedings/proceedings-of-issi-2019.

The paper has now been updated.

