# Supplementary material for: Intermediacy of publications
Source: R Soc Open Sci. 2020 Jan 15;7(1):190207. doi: 10.1098/rsos.190207 (PMC7029947; doi:10.1098/rsos.190207)

Figure S1. Results of main path analysis for case 1. The main path analysis was performed in Pajek using the search path count (SPC) method combined with the standard global search approach. (For each publication, only the name of the first author is shown.)

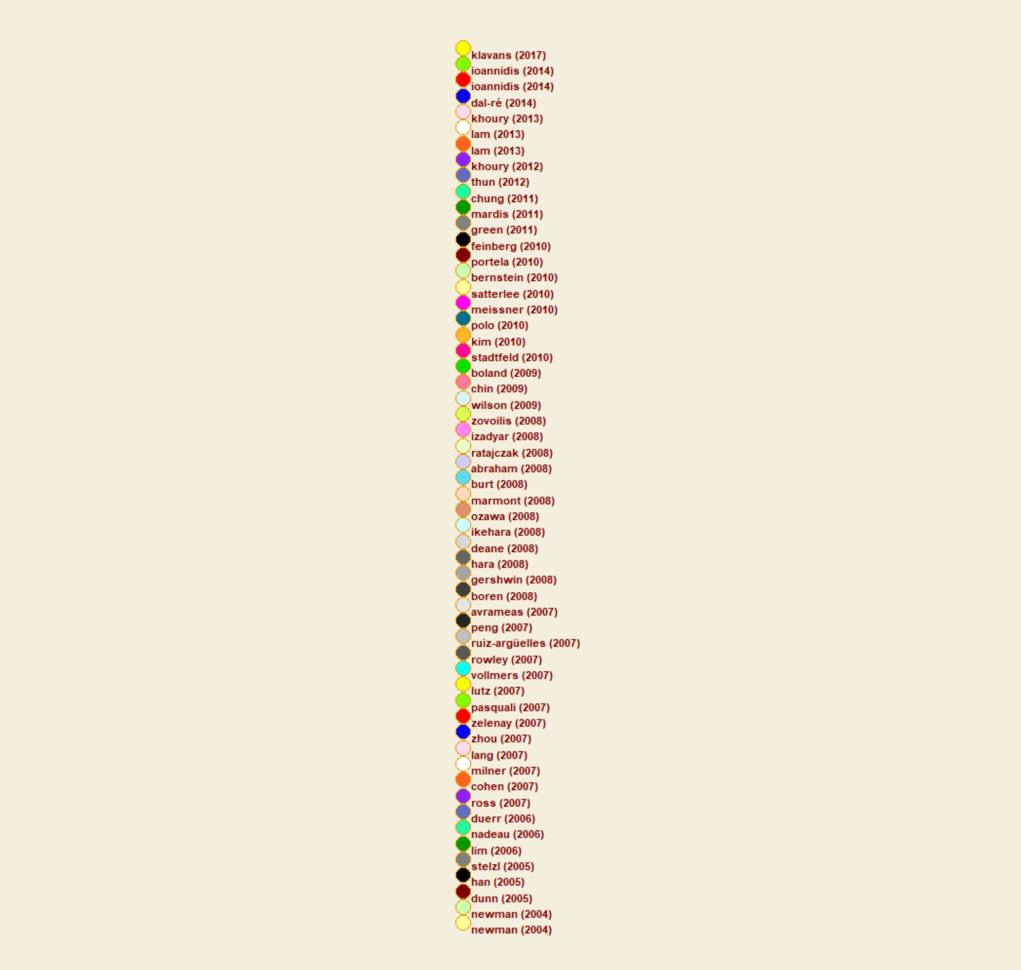

Supplement: Figure S1 [file rsos190207supp1.pdf]
