# Supplementary material for: Intermediacy of publications
Source: R Soc Open Sci. 2020 Jan 15;7(1):190207. doi: 10.1098/rsos.190207 (PMC7029947; doi:10.1098/rsos.190207)

Figure S2. Results of main path analysis for case 1. The main path analysis was performed in Pajek using the search path count (SPC) method combined with the key-route global search approach for the 100 citation links with the highest SPC weights. (For each publication, only the name of the first author is shown.)

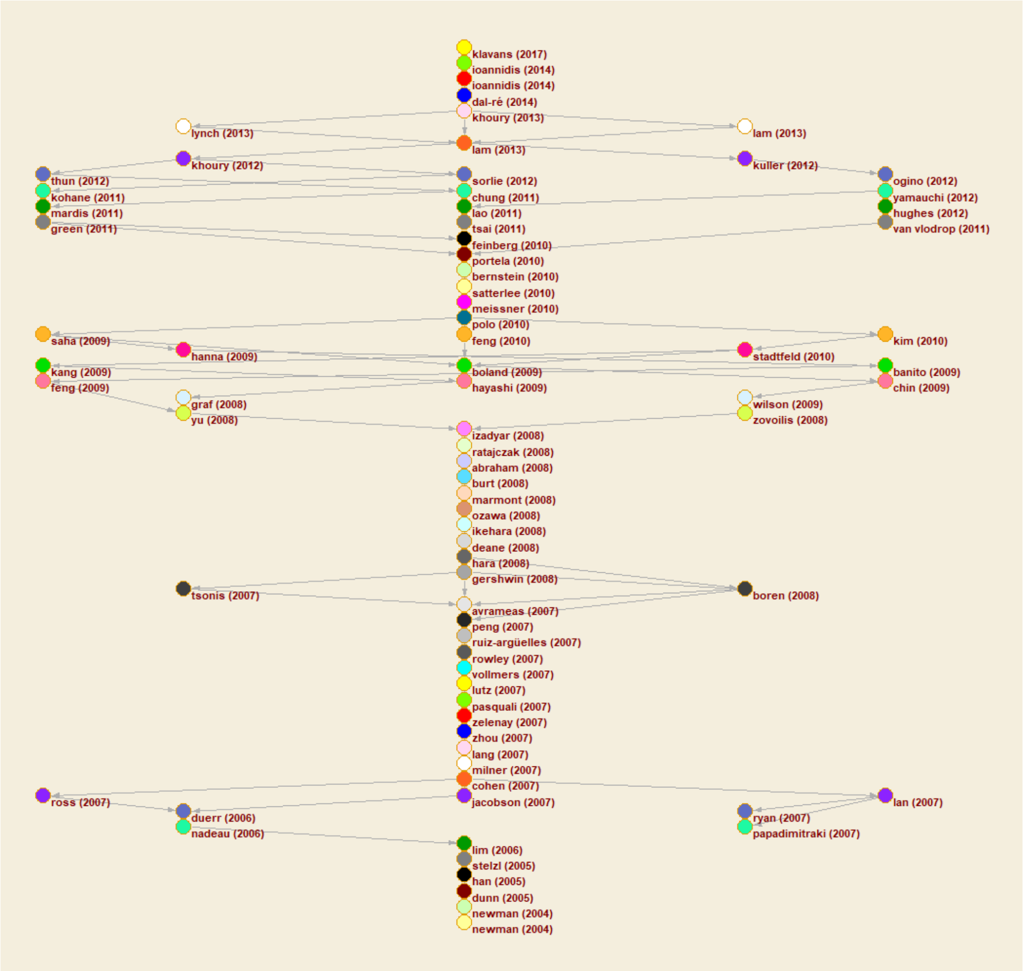

Supplement: Figure S2 [file rsos190207supp2.pdf]
